# Characterizing trajectories of diabetes-related health parameters before diabetes diagnosis in diabetes subtypes: analysis of a 20-year long prospective cohort study in Sweden

**DOI:** 10.1186/s12933-025-02786-6

**Published:** 2025-06-09

**Authors:** Tatjana P. Liedtke, Eike A. Strathmann, Emma Ahlqvist, Olof Asplund, Charlena S. Penz, Paula Stürmer, Cara Övermöhle, Anton Lager, Boel Brynedal, Hrafnhildur Gudjonsdottir, Wolfgang Lieb, Katharina S. Weber

**Affiliations:** 1 Institute of Epidemiology, Kiel University, Niemannsweg 11, 24105 Kiel, Germany; 2 Genetics and Diabetes, Department of Clinical Sciences Malmö, Lund University, Skåne University Hospital, Malmö, Sweden; 3 Center for Epidemiology and Community Medicine (CES), Stockholm, Region Stockholm Sweden; 4 Department of Global Public Health, Karolinska Institutet, Stockholm, Sweden

**Keywords:** Diabetes subtypes, Multilevel modelling, Trajectories, Type 2 diabetes mellitus

## Abstract

**Background:**

Evidence is limited on whether alterations in diabetes-related health parameters are detectable before clinical diagnosis in novel diabetes subtypes. We investigated trajectories of diabetes-related health parameters in individuals with recently diagnosed type 2 diabetes (T2D).

**Methods:**

Using data from the Stockholm Diabetes Prevention Programme cohort (SDPP) participants (n = 215) with recent onset T2D were classified as having severe insulin-deficient diabetes (SIDD, 9%), severe insulin-resistant diabetes (SIRD, 15%), mild obesity-related diabetes (MOD, 14%) and mild age-related diabetes (MARD, 62%). Participants without a family history of diabetes who remained diabetes-free throughout the study served as the controls (n = 2531). Multilevel longitudinal mixed-effects models were used to analyse the trajectories of fasting plasma glucose (FPG) and insulin, body mass index (BMI), homeostasis model assessment estimates of beta-cell function (HOMA2-B) and insulin resistance (HOMA2-IR), waist-to hip-ratio (WHR), diastolic blood pressure (DBP) and systolic blood pressure (SBP) up to 20 years before and 10 years after T2D diagnosis. Pairwise comparisons of the estimated marginal means were used to assess differences between all groups.

**Results:**

Individuals with SIDD consistently exhibited the highest FPG concentrations (*P* < 0.001) and the steepest decline in HOMA2-B levels among all subtypes. BMI was higher in MOD and SIRD than in SIDD and MARD throughout the study period (*P* < 0.01). Individuals with SIRD showed the highest fasting insulin concentrations and higher HOMA2-IR than those with MOD and MARD (*P* < 0.001). WHR and DBP were comparable between subgroups, while SIDD had higher SBP than MOD (*P* = 0.03). The control group exhibited the mildest trajectories across all parameters except for HOMA2-B. Notably, these changes were visible up to 20 years prior to diagnosis.

**Conclusions:**

In a Swedish population, trajectories of diabetes-related health parameters differed up to 20 years before diagnosis between the T2D-related subtypes and controls. This might support early prediction of subtype-specific risks for long-term complications, allowing early initiation of personalized treatment strategies.

**Graphical abstract:**

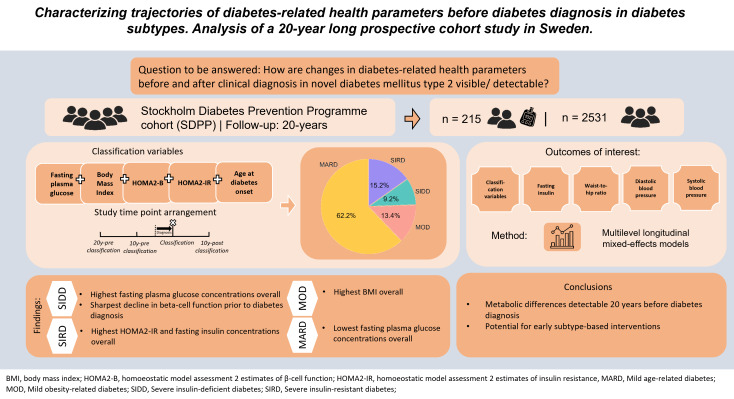

**Supplementary Information:**

The online version contains supplementary material available at 10.1186/s12933-025-02786-6.

## Research Insights

**What is currently known about this topic?**

- To better understand the complexity in clinical features of type 2 diabetes, novel diabetes subtypes were proposed. Few studies researched the long-term trajectories of relevant diabetes-related health parameters in novel diabetes subtypes.

**What is the key research question?**

- How do trajectories of diabetes-related health parameters change before and after the clinical diagnosis in novel subtypes of type 2 diabetes mellitus?

**What is new?**

- Mild and severe type 2 diabetes subtypes were differentiable before diabetes diagnosis based on trajectories of diabetes-related health parameters. Individuals with mild age-related diabetes exhibited the most favourable diabetes-related health parameter trajectories. For several diabetes-related health parameters, subtype trajectories partially shifted in slope and direction after diabetes diagnosis.

**How might this study influence clinical practice?**

- Our findings might help predict subtype-specific risks of long-term complications and therefore support the redefinition of personalized treatment strategies for the type 2 diabetes-related diabetes subtypes.

## Introduction

The global prevalence of diabetes mellitus is rising with significant health implications for those affected [1]. Traditional classifications of diabetes distinguish mainly between individuals with type 1 (T1D) and type 2 diabetes (T2D), who differ in the underlying pathophysiology, clinical manifestations and disease progression. Recently, a new classification scheme has been suggested, in which particularly T2D is no longer regarded as a single entity [2–4]. Based on cluster analysis of six variables at the time of disease diagnosis, Ahlqvist et al*.* [2] distinguished three severe (*i. e.* severe autoimmune diabetes (SAID), severe insulin-deficient diabetes (SIDD), severe insulin-resistant diabetes (SIRD)) and two mild diabetes subtypes (*i. e.* mild obesity-related diabetes (MOD), mild age-related diabetes (MARD)). SAID is defined by glutamic decarboxylase autoantibodies, thus mainly overlaps with T1D. SIDD involves pancreatic beta cell dysfunction, leading to a pronounced lack of insulin secretion. SIRD features a high degree of insulin resistance, MOD is identified by a high body mass index (BMI) and MARD is characterized by later disease onset [2–4]. While previous research within the Whitehall cohort [5] has demonstrated changes in glycemia, insulin sensitivity and insulin secretion became apparent years before the clinical diagnosis and prior to the onset of T2D, it remains unclear whether different trajectories in diabetes-related health parameters exist across T2D-related diabetes subtypes [2]. Investigating these subtype-specific patterns could enhance the understanding of heterogeneous disease progression and improve early risk stratification.

We conducted an analysis within the Stockholm Diabetes Prevention Programme cohort (SDPP) in which we classified individuals into the predefined T2D-related subtypes maximally 5 years after diagnosis. We then aimed to examine the trajectories of diabetes-related health parameters. Trajectories were analyzed in individuals with recent onset T2D up to 20 years before the time of classification and up to 10 years after the time of classification and in individuals who remained free of T2D over the course of the study period.

## Research design and methods

### Study sample and data source

The SDPP is a population-based, prospective cohort study comprising individuals aged 35–56 years who lived in selected municipalities in Stockholm County. A randomly selected sample of participants without a family history of diabetes (FHD) was excluded in order to achieve a balanced ratio between participants with and without FHD. A sub-sample without T2D diagnosis was invited to a clinical examination at baseline (1992–1998), where 3128 men (52% with FHD) and 4821 women (54% with FHD) participated (Exam 1). A follow-up examination (Exam 2) was conducted 10 years later (2002–2008), attended by 2383 men (response rate, 76%) and 3329 women (response rate, 69%). From 2014 to 2017, 20 years after the baseline examination, the third examination (Exam 3) was conducted, with response rate of 57% for men (n = 1752) and 53% for women (n = 2545). SDPP is linked to regional and national registries via participants’ unique personal identity numbers, enabling linkage of new-onset T2D diagnoses from secondary data sources to the data from the different examination cycles. The detailed methodology has been described elsewhere [6].

### Definition of classification time point and pre- and post-classification periods

We aimed to explore the trajectories of diabetes-related health parameters in the years prior to the T2D diagnosis. Therefore, we only included individuals with a diagnosis after the baseline exam 1, so that sufficient data prior to the diagnoses were available.

Since T2D can manifest at any time between the exams, allocation to subtypes was based on data from the first SDPP examination cycle after the diagnosis (this could be either exam 2 or exam 3).

**Fig. 1:**
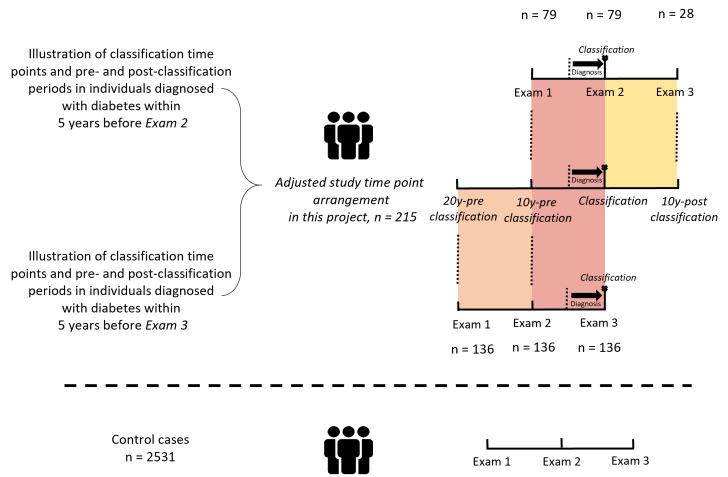
Schematic illustration of the time point of classification and the pre- and post-classification time period in relation to the SPPD exams. 10y-pre classification, 10 years before classification; 20y-pre classification, 20 years before classification; 10y-post classification, 10 years after classification; SDPP, Stockholm Diabetes Prevention Programme. For the study period adjustment, the closest SDPP examination time point to the T2D diagnosis (maximum 5 years before) was defined as “classification”, which was either exam 2 or exam 3. Based on this relative diagnosis time point, the other relative time points were defined. For the control group, the original time points were retained

This strategy was chosen as the classification approach for the subtypes was developed for people with a recent diabetes diagnosis [2]. We allowed a maximum of 5 years between the diagnosis and the SDPP exam used for classification. Next, we defined time periods in relation to the time point of classification. Specifically, we defined the time periods before and after classification as follows: in individuals with exam 2 as the time point of classification, the period was up to 10 years long (10y-pre classification) and in individuals with a diabetes diagnosis close to exam 3, up to 20 years (20y-pre classification). Similarly, the time period after the classification exam could extend up to 10 years (10y-post classification) in those with diagnosis around exam 2 (Fig. 1).

For the present analysis, 215 individuals with incident T2D were diagnosed at or within a maximum of five years before exam 2 (n = 79) or exam 3 (n = 136), respectively and their pre- and post-classification data were pooled for the trajectory analyses. For 28 of the 79 diabetes cases diagnosed at or within 5 years before the exam 2, data was also available for exam 3. As reference sample, we chose 2531 individuals who remained free of incident T2D over the course of the study. We excluded participants with a diagnosis, that was made more than five years before exam 2 or 3 or after exam 2 or 3 (n = 859), participants with missing data (n = 134), participants with hypoglycemia, *i. e.* fasting plasma glucose (FPG) levels < 70 mg/dL (n = 18) and extreme outliers (> 5 standard deviation from the mean; n = 1) from analysis (Supplementary Fig. 1).

Importantly, comparability across examinations is ensured as participants underwent similar examinations at all three time points following standard operating procedures. The SDPP is conducted according to the Declaration of Helsinki. The study protocol has been approved by the ethics board of Swedish Ethics Review Authority (2013/1982-31/2, 2018/2345-32, 2019–06531) and all participants gave their informed consent.

### Definition of diabetes and family history of diabetes

In SDPP, T2D was determined from oral glucose tolerance test (OGTT). The diagnosis was ascertained with fasting plasma glucose ≥ 126 mg/dL and/or 2-h post load plasma glucose ≥ 200 mg/dL [7, 8]. Self-reported diagnoses were collected only at the examination time point and refer to diagnoses occurring since the previous examination. For those who dropped out, diagnostic information was obtained from registry data as code E11 according to the International Classification of Diseases 10th revision as the entire study population was linked to the National Diabetes Register and the Stockholm Regional Healthcare Data Warehouse after the last clinical follow-up in 2017. A positive FHD was defined as having at least one first-degree relative (*e. g.* a parent, sibling or child) or two second-degree relatives (*e. g.* grandparents, aunts or uncles) diagnosed with T2D [6].

### Variables defining the diabetes subtypes (classification variables)

Age at diabetes onset was assessed using a self-administered questionnaire or set to the date of the OGTT examination at which T2D was first diagnosed. Body mass index was calculated from height (measured without shoes) and weight (measured in light indoor clothing [6]. At exam 1 and 2, FPG levels were determined using the glucose oxidase method on a Vitros GLU Slide analyser (Johnson & Johnson Clinical Diagnostics Inc., Rochester, NY, USA) [9]. At exam 3, FPG concentrations were measured in serum and plasma samples using the SYNCHRON LX® and UniCel® DxC 600/800 systems with GLUCm reagent (Beckman Coulter, USA) [10, 11]. Fasting insulin concentrations were measured using a radioimmune-assay. Homeostasis model assessment estimates of beta-cell function (HOMA2-B) and insulin resistance (HOMA2-IR) were calculated using the HOMA2-Calculator Version 2.2.3 of the University of Oxford, using FPG and fasting insulin (University of Oxford, Oxford, UK) [12].

### Outcome definition

As outcomes we chose the subtype-defining variables (*i. e.* FPG, HOMA2-B, HOMA2-IR, BMI) and additional diabetes-related health parameters (*i. e.* fasting insulin, waist-to-hip ratio (WHR), diastolic blood pressure (DBP), systolic blood pressure (SBP)). WHR was measured in supine position. DBP and SDP were measured with an aneroid sphygmomanometer in a sitting position on the left or right arm after a period of rest [13].

### Statistical methodology

Statistical analyses were performed in R (version 4.3.1) and R studio (build 524) [14]. Results are presented as means and standard deviation for normally distributed data and median and interquartile ranges for non-normally distributed continuous data. Kruskal–Wallis tests, followed by *post-hoc* Dunn’s tests, were used to assess differences among the subtypes. Moreover, ANOVAs with *post-hoc* Dunnett’s tests were used to evaluate differences between the subtypes and the controls.

#### Classification approach

The classification methodology by Ahlqvist and colleagues [2] uses BMI [kg/m^2^], age at onset [years], HOMA2-B [%], HOMA2-IR [a. u.], haemoglobin 1Ac (HbA1c) [%] and presence of GADA [yes/no] to categorize individuals with diabetes into one of the five predefined subtypes (clusters). While individuals with positive GADA were assigned to the SAID cluster, the remaining four subtypes (*i. e.* SIDD, SIRD, MOD, MARD) were allocated using the nearest centroid approach [2]. As HbA1c was not available at all examination time points SDPP, the study group led by Emma Ahlqvist developed a modification of their original classification approach based on the ANDIS cohort [2] for this project, in which FPG replaced HbA1c in the centroids. Test accuracy was examined by classifying 9,607 individuals of a replication cohort using both, the original and the new classification approaches (overall probability 88.3%) and by applying the same methodology but excluding both, HbA1c and FPG to evaluate the improvement in classification accuracy achieved by including vs. excluding FPG (overall probability 91.2%).

As the SDPP only studies the development of T2D but not T1D, the SAID cluster (which corresponds closely to T1D) was excluded from analyses.

#### Trajectories of diabetes-related health parameters during the study period

To analyse the trajectories of the diabetes-related health parameters of interest (*i. e.* FPG, BMI, HOMA2-B, HOMA2-IR, fasting insulin, WHR, DBP, SBP) during the study period, a multilevel longitudinal mixed-effects model was used (R package *lme4,* function: *lmer*) [15]. The trajectories were estimated for the four subtypes and the controls. The model accounts for both, fixed effects and individual-specific random effects, addressing intra- and inter-individual variability. To control for potential confounding factors, model 1 was adjusted for the examination cycle, age and sex. Model 2 was additionally adjusted for education, BMI (excluding models with BMI as outcome), smoking behaviour, blood pressure medication (only for models with blood pressure as outcome), FHD and physical activity. The subtype was the grouping variable in all models. To assess differences in the dependent variable in the multilevel longitudinal mixed-effects model between the subtypes, pairwise comparisons of the estimated marginal means were performed.

## Results

### Comparison of the incident diabetes cases with the control group

Compared to those participants who remained free of T2D (controls), participants with incident T2D had a greater share of FHD (81.4% vs. 56.3%), had higher FPG concentrations (median of 130.0 vs. 82.9 mg/dL) and a higher BMI (median 29.4 kg/m^2^ vs. 24.5 kg/m^2^) at exam 1 (Table 1). Participants of both groups had a comparable median age at exam 1 (48 years) (Supplementary Table 1).

**Table 1:** Basic characteristics of individuals with incident type 2 diabetes at exam 2 or 3 (closest time point to T2D diagnosis) and individuals remaining free of type 2 diabetes over the study period (controls) at exam 1

	Incident T2D cases (N = 215)	Individuals remaining free of T2D (N = 2531)	Total (N = 2746)	*P*
Family history of diabetes [n (%)]				
Yes	175 (81.4%)	1425 (56.3%)	1600 (58.3%)	0.0001
No	40 (18.6%)	1042 (41.2%)	1082 (39.4%)	
Missing	0 (0%)	64 (2.5%)	64 (2.3%)	
Diabetes subtypes [n (%)]				
Severe insulin-deficient diabetes (SIDD)	20 (9.3%)	–	–	–
Severe insulin-resistant diabetes (SIRD)	33 (15.3%)	–	–	
Mild obesity-related diabetes (MOD)	29 (13.5%)	–	–	
Mild age-related diabetes (MARD)	133 (61.9%)	–	–	
Age [years]	65.0 [60.0,69.0]	48.0 [44.0,51.0]	48.0 [44.0,52.0]	–
Age [years] at diabetes diagnosis	63.0 [59.0,67.0]	–	–	–
Sex assigned at birth [n (%)]				
Female	94 (43.7%)	1542 (60.9%)	1636 (59.6%)	0.0001
Male	121 (56.3%)	989 (39.1%)	1110 (40.4%)	
BMI [kg/m^2^]	29.4 [26.1,32.9]	24.5 [22.6,26.6]	24.7 [22.8,27.1]	0.0001
BMI categories according to WHO [n (%)]				
Underweight (< 18.5)	0 (0%)	10 (0.4%)	10 (0.4%)	0.0001
Normal weight (18.5–24.9)	41 (19.1%)	1427 (56.4%)	1468 (53.5%)	
Pre-obesity (25–29.9)	74 (34.4%)	931 (36.8%)	1005 (36.6%)	
Obesity class I (30–34.9)	59 (27.4%)	140 (5.5%)	199 (7.2%)	
Obesity class II (35–39.9)	26 (12.1%)	21 (0.8%)	47 (1.7%)	
Obesity class II (> 40)	15 (7.0%)	2 (0.1%)	17 (0.6%)	
Waist to hip ratio	0.95 [0.91, 0.99]	0.83 [0.77, 0.89]	0.84 [0.78,0.90]	0.0001
Fasting plasma glucose concentration [mg/dL]	130 [115, 151]	82.9 [77.5, 88.3]	82.9 [77.5,90.1]	0.0001
Plasma glucose concentration after 2 h [mg/dL]	207 [155, 258]	79.3 [66.7, 91.9]	79.3 [66.7,93.7]	0.0001
Missing	134 (62.3%)	0 (0%)	134 (4.9%)	
Fasting insulin concentration [mIE/L]	17.0 [11.0, 26.0]	12.0 [9.00, 17.0]	12.0 [9.00,17.0]	0.0001
HOMA2-B [%]	76.9 [57.2, 106]	151 [121, 192]	147 [115,189]	0.0001
HOMA2-IR	2.40 [1.50, 3.60]	1.50 [1.10, 2.10]	1.50 [1.10,2.20]	0.0001
Smoking [n (%)]				
Current	18 (8.4%)	493 (19.5%)	511 (18.6%)	0.0001
Former	118 (54.9%)	957 (37.8%)	1075 (39.1%)	
Never	79 (36.7%)	1081 (42.7%)	1160 (42.2%)	
Education [n (%)]				
Blue collar worker, unskilled	31 (14.4%)	115 (4.5%)	146 (5.3%)	0.0001
Blue collar worker, skilled	31 (14.4%)	235 (9.3%)	266 (9.7%)	
White collar worker, lower	65 (30.2%)	746 (29.5%)	811 (29.5%)	
White collar worker, middle	36 (16.7%)	444 (17.5%)	480 (17.5%)	
White collar worker, higher	32 (14.9%)	474 (18.7%)	506 (18.4%)	
Self-employed	20 (9.3%)	498 (19.7%)	518 (18.9%)	
Others, not classified	0 (0%)	19 (0.8%)	19 (0.7%)	
Deceased [n (%)]				
Yes	31 (14.4%)	93 (3.7%)	124 (4.5%)	0.0001
No	184 (85.6%)	2438 (96.3%)	2622 (95.5%)	
Physical activity [n (%)]				
Sedentary	25 (11.6%)	229 (9.0%)	254 (9.2%)	0.0594
Light physical activity	111 (51.6%)	1273 (50.3%)	1384 (50.4%)	
Moderate physical activity	53 (24.7%)	809 (32.0%)	862 (31.4%)	
Moderate to vigouros physical activity	26 (12.1%)	220 (8.7%)	246 (9.0%)	

Data are presented as means ± SD for normally distributed data and median (Q25; Q75) for non-normally distributed continuous data. Means and medians of the two samples were compared using Student’s t-test (equal variances) or Welch’s t-test (unequal variances) for normally distributed continuous variables or Mann–Whitney-U-test for continuous non-normally distributed variables, respectively. To compare categorical variables the chi-square test and Fisher’s exact test were used

T2D, type 2 diabetes; BMI, body mass index; HOMA2-B, homoeostatic model assessment 2 estimates of β-cell function; HOMA2-IR, homoeostatic model assessment 2 estimates of insulin resistance

### Comparison of the defining characteristics of the diabetes subtypes

Of the 215 participants developing incident T2D during the observations period, 20 were assigned to the SIDD, 33 to the SIRD, 29 to the MOD and 133 to the MARD subtype. The median age at diabetes diagnosis was 63 years (Table 1).

At the time point of classification, the SIDD compared to the remaining subtypes was characterized by elevated FPG concentrations, lower HOMA2-B and a lower BMI (Supplementary Fig. 2). In contrast, the SIRD subtype was characterized by higher HOMA2-IR and obesity. The MOD subtype was characterized by obesity and younger age at onset. The MARD subtype was characterized by older age at diabetes onset and a lower BMI in comparison to the remaining subtypes (Table 1, Supplementary Fig. 2).

### Trajectories of diabetes-related health parameters across subtypes

**Fig. 2:**
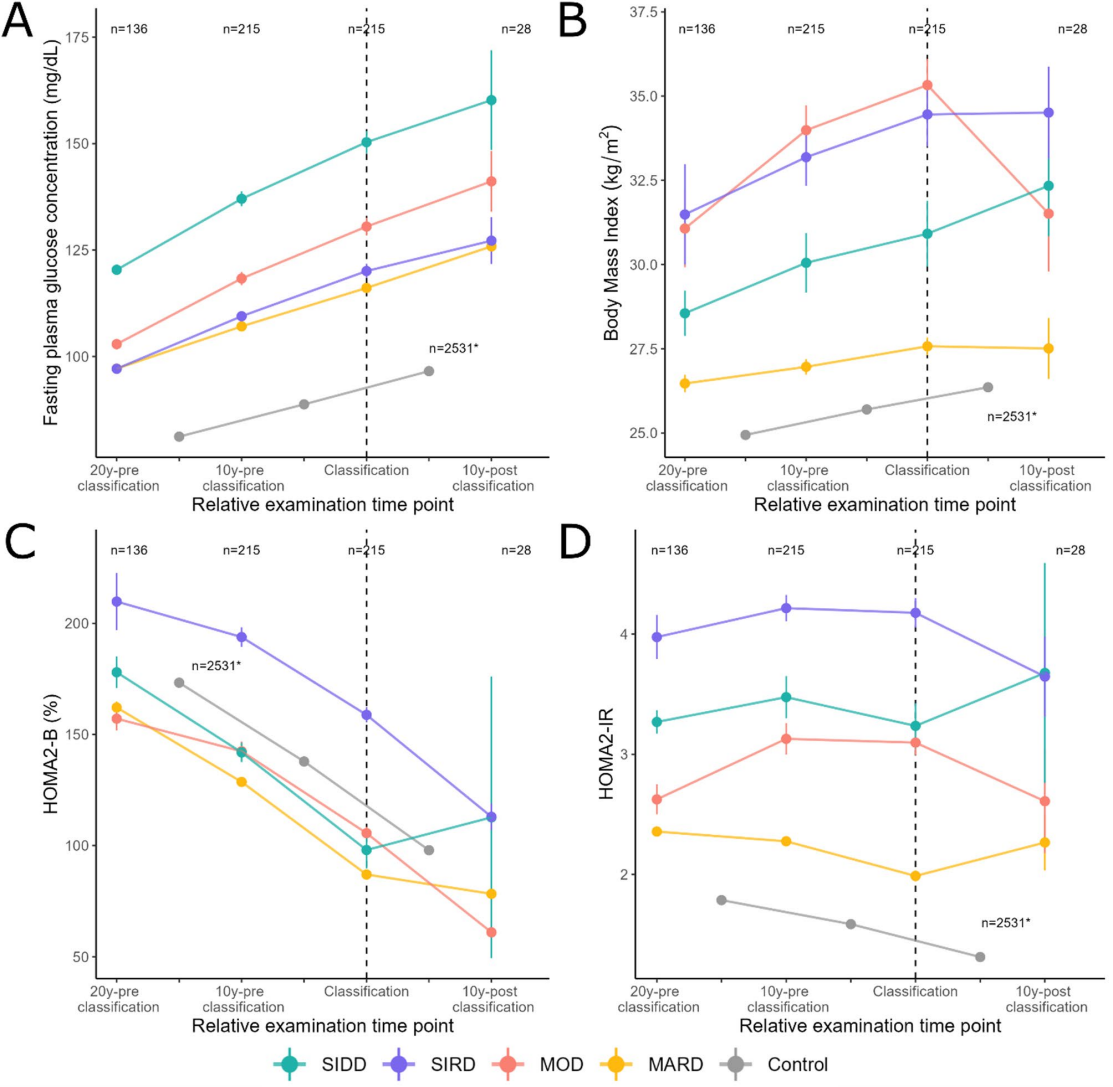
Trajectories of variables defining the diabetes subtypes (classification variables), fully adjusted model 2. Multilevel longitudinal modelling was done for fasting glucose (**A**), body mass index (**B**), HOMA2-B (**C**), HOMA2-IR (**D**) in individuals with incident diabetes (stratified by diabetes subtype) and in individuals without diabetes. * n = 2,531 for all examination time points of the control sample. Graphs show mean and standard error for the fixed effects. The fully adjusted model 2 was adjusted for age, sex, study time point, education, body mass index (except for models with body mass index as the dependent variable), smoking behaviour, family history of diabetes, physical activity and blood pressure medication (only for models with systolic and diastolic blood pressure as the dependent variables). For the definitions of the time point of classification and the pre- and post-classification time periods, please see “Definition of the time point of classification and of the time periods before and after classification” in the methods section for details. 10y-pre classification, 10 years before classification; 20y-pre classification, 20 years before classification; 10y-post classification, 10 years after classification; HOMA2-B, homoeostatic model assessment 2 estimates of β-cell function; HOMA2-IR, homoeostatic model assessment 2 estimates of insulin resistance; MARD, moderate age-related diabetes; MOD, moderate obesity-related diabetes; SIDD, severe insulin-deficient diabetes; SIRD, severe insulin-resistant diabetes; T2D, type 2 diabetes

The FPG concentrations showed an approximately linear increasing trend with time for individuals with SIRD, MOD, MARD and the controls. For SIDD, which had the highest FPG concentrations at all relative examination time points, FPG increased until the classification time point and remained nearly constant during the following 10 years. Controls had the lowest glucose values and the lowest/flattest slope compared to the subtypes. FPG concentrations were intermediate for individuals with SIRD, MOD and MARD. Trajectories differed significantly between subtypes except for MARD compared with SIRD (Fig. 2A, Supplementary Tables 2,4).

Mean BMI values showed an inverse U-shaped trend with the highest BMI at the time of classification in individuals with MOD. Among SIRD (until time point of classification), SIDD and the controls, BMI increased linearly over time. Among MARD, BMI increased only moderately over the observation period. In absolute terms, BMI was highest in individuals with MOD (time point of classification) and SIRD (10 years thereafter), respectively, followed by those with SIDD and MARD and lowest in the controls. Trajectories differed significantly between subtypes except for MOD and SIRD (Fig. 2B, Supplementary Tables 2,4).

HOMA2-B showed a decreasing, approximately linear trend in all subtypes as well as controls. Individuals with SIDD had the largest decline in HOMA2-B values, starting with the second highest mean HOMA2-B value 20 years before classification and falling to the second lowest mean HOMA2-B value at time point of classification, while increasing the following 10 years. Compared to the other four subtypes, those with SIRD had the highest HOMA2-B values at all relative examination time points. Further, their HOMA2-B values fell more sharply from ten years before the classification time point until the end of the observation period than during the first ten years of observation. A comparable trend but with lower absolute HOMA2-B level was observed among individuals with MOD. In contrast, in MARD, the drop in the HOMA2-B value slowed down after the classification time point. Trajectories differed significantly between the subtypes with the exception for SIDD with MOD and SIDD with MARD as well as MARD with MOD (Fig. 2C, Supplementary Tables 2,4).

**Fig. 3:**
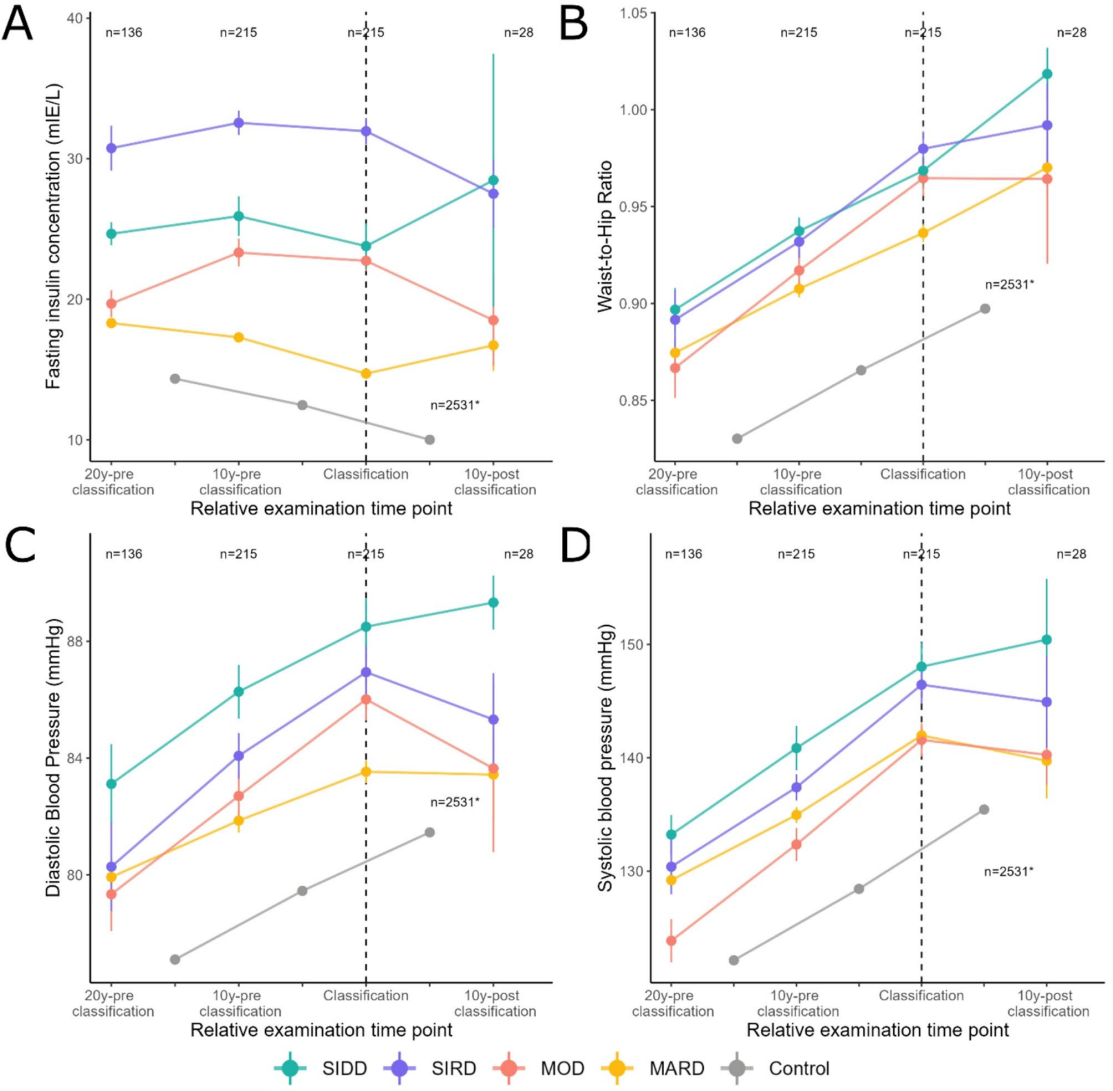
Trajectories of additional diabetes-related health parameters, fully adjusted model 2. Multilevel longitudinal modelling was done for fasting insulin (**A**), waist-to-hip ratio (**B**), diastolic blood pressure (**C**), systolic blood pressure (**D**) in individuals with incident diabetes (stratified by diabetes subtype) and in individuals without diabetes. * n = 2,531 for all examination time points of the control group. Graphs show mean and standard error for the fixed effects. The fully adjusted model 2 was adjusted for age, sex, study time point, education, body mass index (except for models with body mass index as the dependent variable), smoking behaviour, family history of diabetes, physical activity and blood pressure medication (only for models with systolic and diastolic blood pressure as the dependent variables). For the definitions of the time point of classification and the pre- and post-classification time periods, please see “Definition of the time point of classification and of the time periods before and after classification” in the methods section for details. 10y-pre classification, 10 years before classification; 20y-pre classification, 20 years before classification; 10y-post classification, 10 years after classification; HOMA2-B, homoeostatic model assessment 2 estimates of β-cell function; HOMA2-IR, homoeostatic model assessment 2 estimates of insulin resistance; MARD, moderate age-related diabetes; MOD, moderate obesity-related diabetes; SIDD, severe insulin-deficient diabetes; SIRD, severe insulin-resistant diabetes; T2D, type 2 diabetes

Trends in mean values of HOMA2-IR and fasting insulin concentrations were comparable showing an inverse U-shaped trend in individuals with SIRD and MOD, with a decline in insulin concentrations after the classification time point. In SIDD and MARD, the trend was reversed, thus U-shaped, with increasing insulin concentrations from the time after the classification time point. Fasting insulin concentrations were highest in individuals with SIRD and lowest in the controls, in which the concentrations decreased linearly over time. 20 years before the classification time point, concentrations were intermediate in individuals with SIDD and lowest in those with MOD and MARD. 10 years after the classification time point, however, fasting insulin concentrations were comparable between MOD and MARD. Trajectories differed significantly between the subtypes with the exception for MOD with MARD (HOMA2-IR) and MOD with MARD (insulin) (Figs. 2C, 3A, Supplementary Tables 2–5).

The WHR showed an increasing trend for all subtypes, which was linear for individuals with SIDD, MARD and the controls. Among SIRD, the increase of WHR was largest ten years before the classification time point and levelled off afterwards. In individuals with MOD, WHR increased in parallel with those with SIRD in the ten years prior to the classification time point, but remained stable afterwards. In absolute terms, mean WHR was lowest in the controls at all examination time points. At the beginning and the end of the observation period, WHR was intermediate for individuals with MOD and MARD and highest for those with SIDD and SIRD, while at the classification time point, mean WHR of MOD were at the level of those with SIDD and SIRD. Trajectories were comparable between all subtypes (Fig. 3B, Supplementary Tables 3,5).

In individuals with SIDD and in the controls, mean DBP values increased during the whole study period with values being highest at all examination points for SIDD and lowest among controls. In contrast, DBP values in SIRD, MOD and MARD increased before the classification time point but decreased or remained stable thereafter. Trajectories were comparable between all subtypes (Fig. 3C, Supplementary Tables 3,5).

There was an increasing trend for SBP in all subtypes and the controls, with SBP rising most sharply ten years before the classification time point. Thereafter, SBP only continued to rise for those with SIDD, but with a flatter slope. In the remaining individuals, SBP fell slightly thereafter. As for DBP, SBP was highest in individuals with SIDD and lowest in the controls during the whole study period. Trajectories were comparable between all subtypes and only differed between SIDD and MOD (Fig. 3D, Supplementary Tables 3,5). The results of model 1 (Supplementary Figs. 3,4,6,7) were largely consistent with those of the fully adjusted model 2 (Figs. 2,3, Supplementary Tables 2,3).

To determine whether the exclusion of individuals from the control cohort with FHD influenced the results, a sensitivity analysis was performed. The results of the sensitivity analysis were consistent with those of the main analysis (Supplementary Fig. 5,6).

## Discussion

We examined the trajectories diabetes-related health parameters before and after diagnosis in four T2D-related subtypes and in individuals remaining free of diabetes during the observation period. The main observations were as follows: First, changes in trajectories were visible up to 20 years prior to diagnosis. Second, the mild (MOD, MARD) and the severe subtypes (SIDD, SIRD) were distinguishable already before the time point of diagnosis. Third, individuals with MARD showed the most beneficial trajectories for most of the parameters studied. Trajectories for MOD were mainly intermediate between those for MARD and those for the severe subtypes. As expected, trajectories for SIDD were worst for FPG, beta-cell function and blood pressure, while trajectories for SIRD were worst for markers of insulin resistance. Third, for several biomarkers, the trajectories seemed to partially change in slope and direction in the subtypes after the diagnosis has been made.

### In the context of the current literature

#### SIDD

In individuals with SIDD, FPG increased, with second highest FPG concentrations around diabetes diagnosis, both being consistent with previous findings [3]. Prior studies reported not only an association of higher pre-diagnostic plasma glucose levels with an increased risk of developing T2D [16, 17], but additionally with a higher mortality in participants with T2D [18, 19]. Similarly to the results by Tabak et al*.* [5], initial FPG concentrations 20y-pre classification were in the prediabetic range.

As elevated WHR is associated with increased risk of T2D [20–23], the continuous increase in WHR among individuals with SIDD may a indicate the importance of lifestyle interventions reducing WHR.

HOMA2-B levels steadily declined for 20 years prior to diagnosis, which was sharpest for the last 10 years before diagnosis. Our findings build upon the study by Tabak et al. by providing additional insights into the trajectory of beta-cell function [5]. Studies showed a causal association of HOMA2-B with T2D incidence [24] and a lower HOMA2-B was associated with future therapy failure, indicating the need for insulin therapy initiation [25].

#### SIRD

In individuals with SIRD, FPG concentrations increased over the observation period, aligning with the results of Zaharia et al*.* [3]. Furthermore, HOMA2-IR and fasting insulin concentrations were highest throughout the observation period and increased before the time of diagnosis. This is in line with previous literature, where whole-body insulin sensitivity decreased during the first five years after diagnosis in individuals with SIRD [3]. In the Whitehall II study, increases in fasting insulin concentrations were already recognizable up to 20 years before T2D diagnosis [26]. Furthermore, individuals with both insulin deficiency and insulin resistance were at a higher risk for developing T2D and required insulin treatment earlier than those without [27].

#### MOD

Individuals with MOD exhibited an increase in FPG as observed in the study by Zaharia et al*.* [3]. They also displayed the highest mean BMI throughout the study period, with values corresponding to obesity [28] as early as 20y-pre classification. Previous studies indicate that an increased BMI is strongly associated with a higher risk of developing T2D [20, 29–33]. Moreover, a higher BMI prior to diagnosis is linked to increased mortality in individuals with T2D [18], highlighting the long-term implications of excess body weight, especially visible in the MOD subtype.

#### MARD

FPG concentrations increased but remained lowest overall in individuals with MARD after 20y-pre classification, increasing slightly at each examination time point during the observation period. Similarly, a recent study in newly diagnosed individuals with MARD showed only slight increases in FPG during 4.4 years with overall lowest concentrations amongst the subgroups [34]. Furthermore, another study [35] found that individuals with MARD, who were older at diagnosis, exhibit only moderate metabolic abnormalities and only modest derangements in key metabolic parameters such as HbA1c, BMI and C-peptide levels compared to other, more severe T2D subtypes. Thus, despite the slow progression of the disease in this subgroup, the remaining risk emphasizes the importance of T2D treatment [36].

#### Healthy controls

Despite the adjustment for confounders in the multilevel longitudinal mixed-effects model and the exclusion of individuals with FHD, a worsening for most of the parameters was observed in the non-incident controls over the observation period. This might be due to residual confounding by *e. g*. diet, stress or other environmental/ lifestyle changes and the natural effect of aging.

Thus far, approaches to prevent diabetes development are not tailored to specific diabetes phenotypes, but rather apply universally. Known strategies with a demonstrated efficacy to avoid progression from prediabetes to diabetes include physical activity, diet and metformin [37]. Interestingly, mounting observational evidence suggests that the association of (correlates of) physical fitness and diet with cardiovascular risk factors differs between the different diabetes subtypes [38–41]. It is therefore conceivable that a better understanding of the biochemical and metabolic differences between the diabetes subtypes might contribute to more tailored prevention approaches. A concept that warrants further investigation.

### Strengths and limitations

Strengths of our study are, firstly, that T2D diagnosis was based on OGTTs within the SDPP, as it has the highest sensitivity for detecting diabetes. Secondly, the introduction of the subtyping based on FPG instead of HbA1c enables application of the classification approach also in cohorts where HbA1c is not available. Although the overall accuracy of this approach is too low for clinical diagnostic purposes, it can be used effectively for cohort-level statistical analyses where the estimated accuracies on a replication dataset were high. Several limitations merit consideration. First, although the study benefits from a large control sample, the relatively small number of participants with recent onset T2D and their uneven distribution across diabetes subtypes with the MARD group being predominant may limit the generalizability of the findings. Notably, a predominance of the MARD subtype has also been reported in other studies [35, 42, 43]. Additionally, especially at 10y-post classification, small sample sizes within the subgroups increase the risk of type 2 error, potentially leading to an under detection of true associations. In future studies, larger and more balanced cohorts will be necessary to validate these trends. Second, due to the long intervals between the exams, we cannot rule out that participants experienced clinically relevant changes in body weight and other health-related parameters, for which we could not account in the analysis. Third, information on diabetes medication is not available so that *e. g.* adjusting the trajectories after diabetes diagnosis for potential confounding effects is not possible. The improvements in trajectories observed in some subtypes indicate that these individuals received effective therapeutic measures. Forth, deviating from the method of Ahlqvist et al*.* [2], insulin was used instead of C-peptide for classification, as C-peptide was not measured in SDPP. However, as we only included the T2D-related subtypes, we do not expect any limitations by not distinguishing endogenous from exogenous insulin production. Fifth, the follow-up duration of ten years is too long to examine short-term changes. Sixth, study participants of the SDPP are all born in Sweden so that our results are not generalizable to individuals of other geographic origin.

## Conclusion

In conclusion, this study shows that the T2D-related subtypes are distinguishable already before the time of diagnosis based on the trajectories of diabetes-related health parameters and that trajectories seem to partially change after diagnosis. If confirmed in larger and more diverse cohorts with shorter follow-up periods between examination time points, these observations might help predict subtype-specific long-term complication risks already in the prediabetic phase, aiding refined, personalized treatment strategies for the T2D-related subtypes. Also, dynamic classification models should be considered in future research, to account for potential shifts in subtypes. Thus, early intervention efforts for individuals at higher risk for more severe disease outcomes can be improved.

## Electronic supplementary material

Below is the link to the electronic supplementary material.

Supplementary Material 1

## Data Availability

The SDPP data used for this study are available upon reasonable request, appropriate ethical approval and data protection arrangements. Data from the SDPP are protected by the General Data Protection Regulation of the European Union, which prevents open access to the personal data of the study. To obtain deidentified data for research purposes, interested researchers can contact the Centre for Epidemiology and Community Medicine in Stockholm, Sweden (ces.slso@regionstockholm.se). The R scripts are available from the lead author upon reasonable request.

## Abbreviations

BMI: Body mass index
DBP: Diastolic blood pressure
FHD: Family history of diabetes
FPG: Fasting plasma glucose
HOMA2-B: Homeostasis model assessment estimates of beta-cell function
HOMA2-IR: Homeostasis model assessment estimates of insulin resistance
MARD: Mild age-related diabetes
MOD: Mild obesity-related diabetes
OGTT: Oral glucose tolerance test
SBP: Systolic blood pressure
SDPP: Stockholm Diabetes Prevention Programme cohort
SIDD: Severe insulin-deficient diabetes
SIRD: Severe insulin-resistant diabetes
T2D: Type 2 diabetes
WHR: Waist-to hip-ratio
