# Supplementary material for: Characterizing trajectories of diabetes-related health parameters before diabetes diagnosis in diabetes subtypes: analysis of a 20-year long prospective cohort study in Sweden
Source: Cardiovasc Diabetol. 2025 Jun 9;24:244. doi: 10.1186/s12933-025-02786-6 (PMC12150535; doi:10.1186/s12933-025-02786-6)
Supplement: Supplementary file 1 — Supplementary Material 1 [file 12933_2025_2786_MOESM1_ESM.docx]

**SUPPLEMENTARY MATERIAL**

**Supplementary Table 1-** Basic characteristics of individuals with incident type 2 diabetes and individuals remaining free of type 2 diabetes over the study period (controls) at exam 1

|  | **Incident T2D cases (N=215)** | **Individuals remaining free of T2D (N=2531)** | **Total (N=2746)** | **P** |
| --- | --- | --- | --- | --- |
| **Family history of diabetes [n (%)]** | | | | |
| Yes | 175 (81.4%) | 1425 (56.3%) | 1600 (58.3%) | 0.0001 |
| No | 40 (18.6%) | 1042 (41.2%) | 1082 (39.4%) |  |
| Missing | 0 (0%) | 64 (2.5%) | 64 (2.3%) |  |
| **Diabetes subtypes [n (%)]** | | | | |
| Severe insulin-deficient diabetes (SIDD) | 20 (9.3%) | - | - | - |
| Severe insulin-resistant diabetes (SIRD) | 33 (15.3%) | - | - |  |
| Mild obesity-related diabetes (MOD) | 29 (13.5%) | - | - |  |
| Mild age-related diabetes (MARD) | 133 (61.9%) | - | - |  |
| **Age [years]** | 48.0 [45.0,51.0] | 48.0 [44.0,51.0] | 48.0 [44.0,51.0] | 0.0372 |
| **Age [years] at diabetes diagnosis** | 63.0 [59.0,67.0] | - | - | - |
| **Sex assigned at birth [n (%)]** | | | | |
| Female | 94 (43.7%) | 1542 (60.9%) | 1636 (59.6%) | 0.0001 |
| Male | 121 (56.3%) | 989 (39.1%) | 1110 (40.4%) |  |
| **BMI [kg/m^2^]** | 27.5 [25.1,30.1] | 24.5 [22.6,26.6] | 24.6 [22.8,27.0] | 0.0001 |
| **BMI categories according to WHO [n (%)]** | | | | |
| Underweight (<18.5) | 0 (0%) | 10 (0.4%) | 10 (0.4%) | 0.0001 |
| Normal weight (18.5 - 24.9) | 53 (24.7%) | 1427 (56.4%) | 1480 (53.9%) |  |
| Pre-obesity (25-29.9) | 106 (49.3%) | 931 (36.8%) | 1037 (37.8%) |  |
| Obesity class I (30-34.9) | 36 (16.7%) | 140 (5.5%) | 176 (6.4%) |  |
| Obesity class II (35-39.9) | 14 (6.5%) | 21 (0.8%) | 35 (1.3%) |  |
| Obesity class II (>40) | 6 (2.8%) | 2 (0.1%) | 8 (0.3%) |  |
| **Waist to hip ratio** | 0.90 [0.85,0.93] | 0.83 [0.77,0.885] | 0.83 [0.78,0.89] | 0.0001 |
| **Fasting plasma glucose concentration [mg/dL]** | 93.7 [86.5,101] | 82.9 [77.5,88.3] | 82.9 [77.5,88.3] | 0.0001 |
| **Plasma glucose concentration after 2 h [mg/dL]** | 103 [82.9,128] | 79.3 [66.7,91.9] | 79.3 [67.1,95.1] | 0.0001 |
| **Fasting insulin concentration [mIE/L]** | 18.0 [14.0,25.0] | 12.0 [9.00,17.0] | 12.0 [9.00,18.0] | 0.0001 |
| **HOMA2-B [%]** | 157 [126,201] | 151 [121,192] | 152 [121,192] | 0.2049 |
| **HOMA2-IR** | 2.40 [1.80,3.10] | 1.50 [1.10,2.10] | 1.60 [1.10,2.20] | 0.0001 |
| **Smoking [n (%)]** | | | | |
| Current | 62 (28.8%) | 493 (19.5%) | 555 (20.2%) | 0.0001 |
| Former | 90 (41.9%) | 957 (37.8%) | 1047 (38.1%) |  |
| Never | 63 (29.3%) | 1081 (42.7%) | 1144 (41.7%) |  |
| **Education [n (%)]** | | | | |
| Blue collar worker, unskilled | 33 (15.3%) | 115 (4.5%) | 148 (5.4%) | 0.0001 |
| Blue collar worker, skilled | 31 (14.4%) | 235 (9.3%) | 266 (9.7%) |  |
| White collar worker, lower | 66 (30.7%) | 746 (29.5%) | 812 (29.6%) |  |
| White collar worker, middle | 34 (15.8%) | 444 (17.5%) | 478 (17.4%) |  |
| White collar worker, higher | 32 (14.9%) | 474 (18.7%) | 506 (18.4%) |  |
| Self-employed | 19 (8.8%) | 498 (19.7%) | 517 (18.8%) |  |
| Others, not classified | 0 (0%) | 19 (0.8%) | 19 (0.7%) |  |
| **Deceased [n (%)]** | | | | |
| Yes | 31 (14.4%) | 93 (3.7%) | 124 (4.5%) | 0.0001 |
| No | 184 (85.6%) | 2438 (96.3%) | 2622 (95.5%) |  |
| **Physical activity [n (%)]** | | | | |
| Sedentary | 30 (14.0%) | 229 (9.0%) | 259 (9.4%) | 0.0018 |
| Light physical activity | 124 (57.7%) | 1273 (50.3%) | 1397 (50.9%) |  |
| Moderate physical activity | 50 (23.3%) | 809 (32.0%) | 859 (31.3%) |  |
| Moderate to vigouros physical activity | 11 (5.1%) | 220 (8.7%) | 231 (8.4%) |  |

**Supplementary Figure 1**- Flowchart showing the Stockholm Diabetes Prevention Programme cohort being eligible for analysis


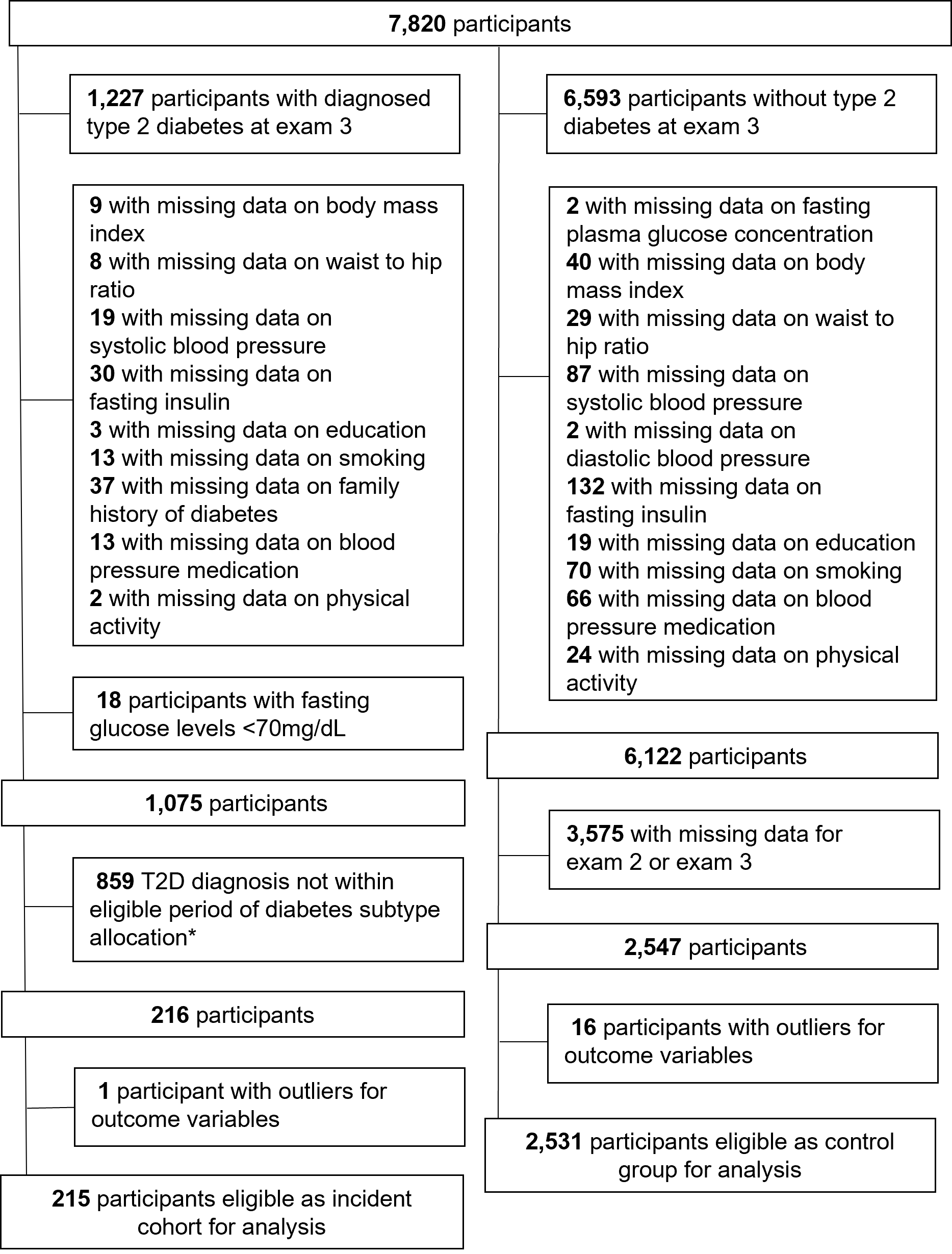


*maximum 5 years before exam 2 or exam 3.

T2D, type 2 diabetes.

**Supplementary Figure 2**- Variables defining the diabetes subtypes (classification variables) and additional diabetes-related health parameters in individuals with type 2 diabetes at the time of classification and in the control cohort at exam 1


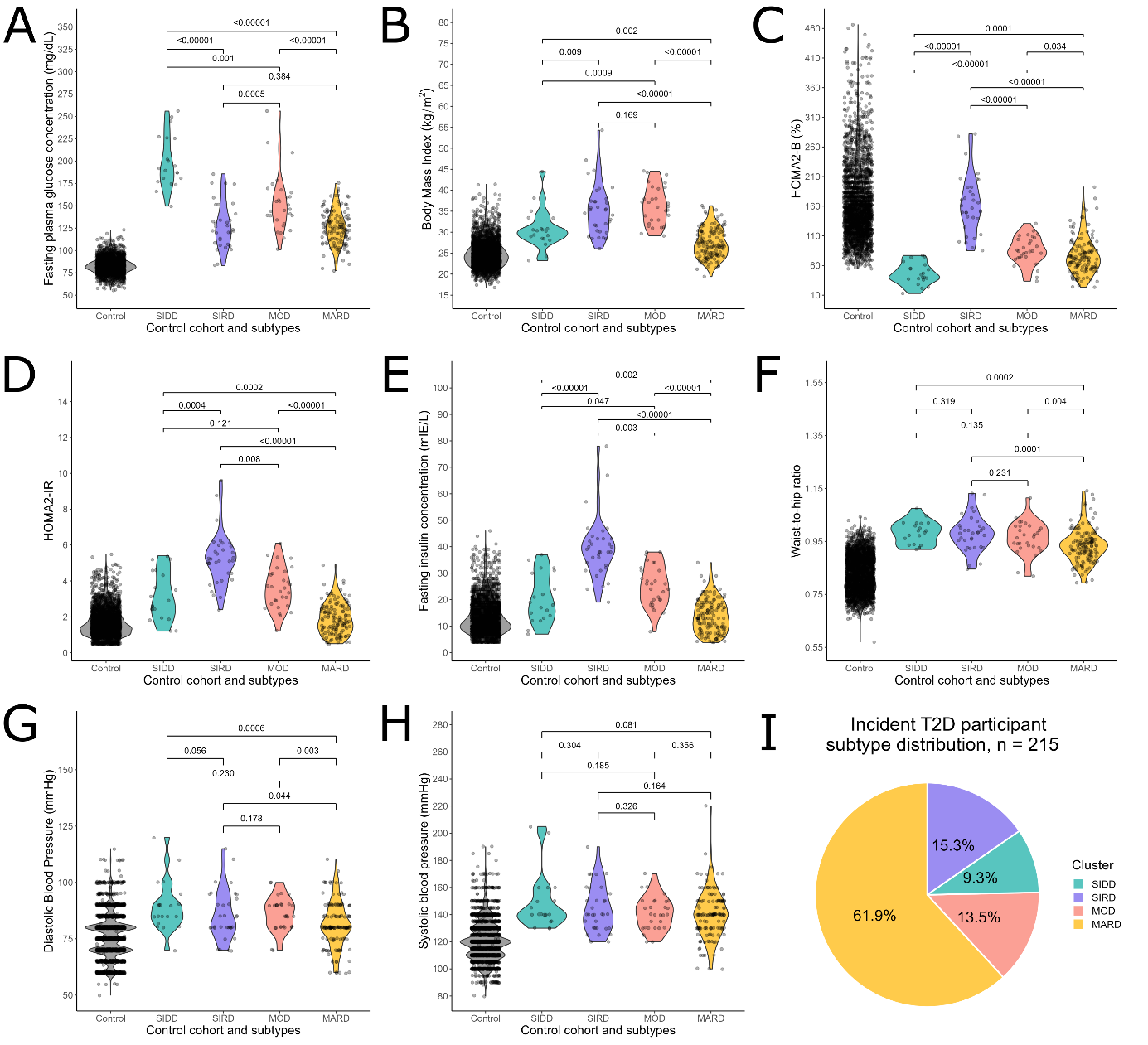


Data (A:H) are median and interquartile range. Group comparisons were performed between the subtypes using the Kruskal-Wallis test, followed by *post-hoc* Dunn's test to calculate p-values for pairwise comparisons. (I) shows the subtype distribution in the incident population.

HOMA2-B, homoeostatic model assessment 2 estimates of β-cell function; HOMA2-IR, homoeostatic model assessment 2 estimates of insulin resistance; MARD, moderate age-related diabetes; MOD, moderate obesity-related diabetes; SIDD, severe insulin-deficient diabetes; SIRD, severe insulin-resistant diabetes; T2D, type 2 diabetes.

**Supplementary Figure 3-** Trajectories of variables defining the diabetes subtypes (classification variables), Model 1

**
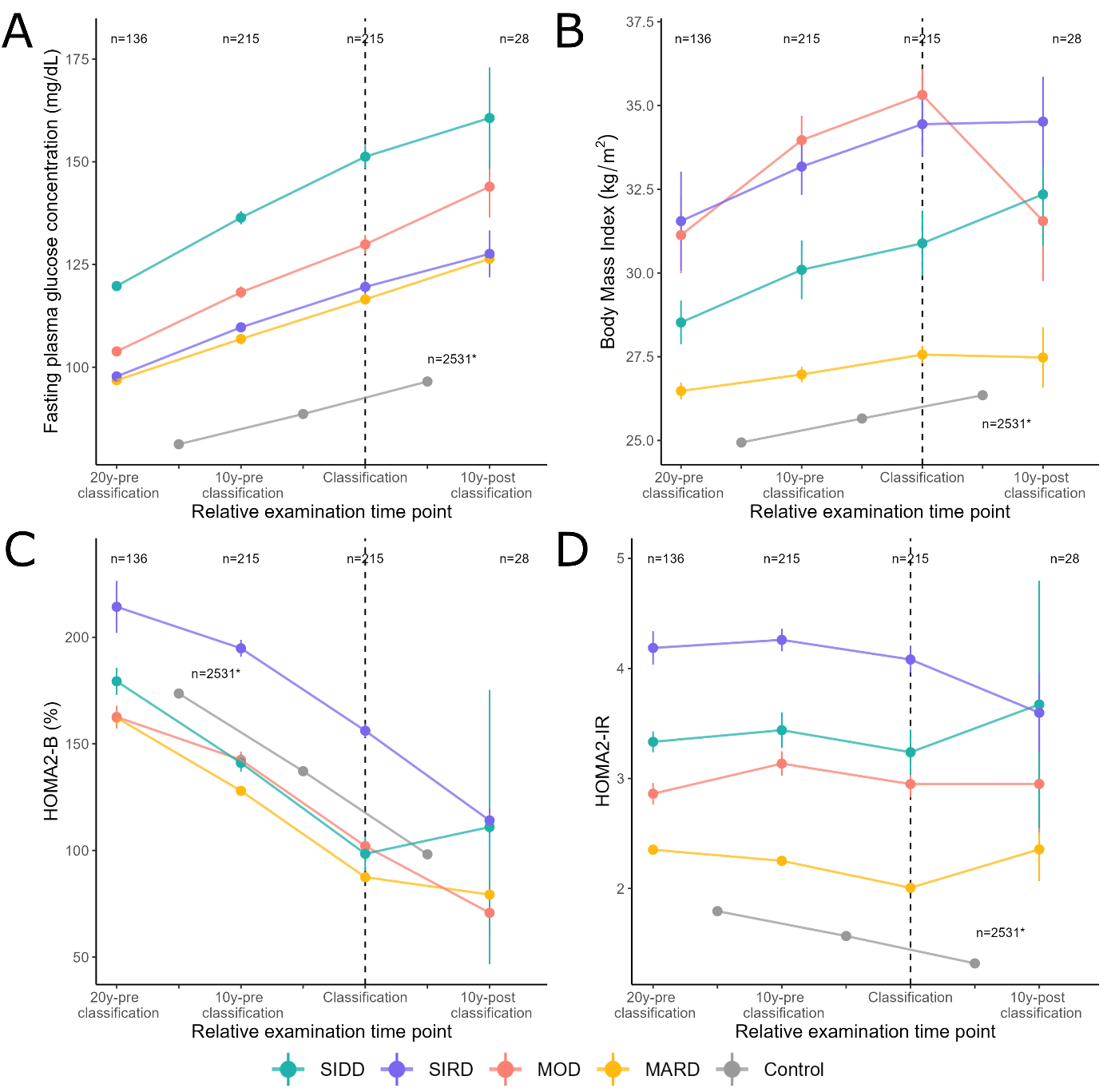
**

Multilevel longitudinal modelling was done for fasting glucose (A), body mass index (B), HOMA2-B (C), HOMA2-IR (D) in individuals with incident diabetes (stratified by diabetes subtype) and in individuals without diabetes.

* n=2,548 for all examination time points of the control sample.

Analysis was adjusted for age, sex and examination time point.

Graph shows mean and standard error for the fixed effects.

For the definitions of the time point of classification and the pre- and post-classification time periods, please see “Definition of the time point of classification and of the time periods before and after classification” in the methods section for details.

10y-pre classification, 10 years before classification; 20y-pre classification, 20 years before classification; 10y-post classification, 10 years after classification; HOMA2-B, homoeostatic model assessment 2 estimates of β-cell function; HOMA2-IR, homoeostatic model assessment 2 estimates of insulin resistance; MARD, moderate age-related diabetes; MOD, moderate obesity-related diabetes; SIDD, severe insulin-deficient diabetes; SIRD, severe insulin-resistant diabetes; T2D, type 2 diabetes.

**Supplementary Figure 4-** Trajectories of additional diabetes-related health parameters, Model 1

**
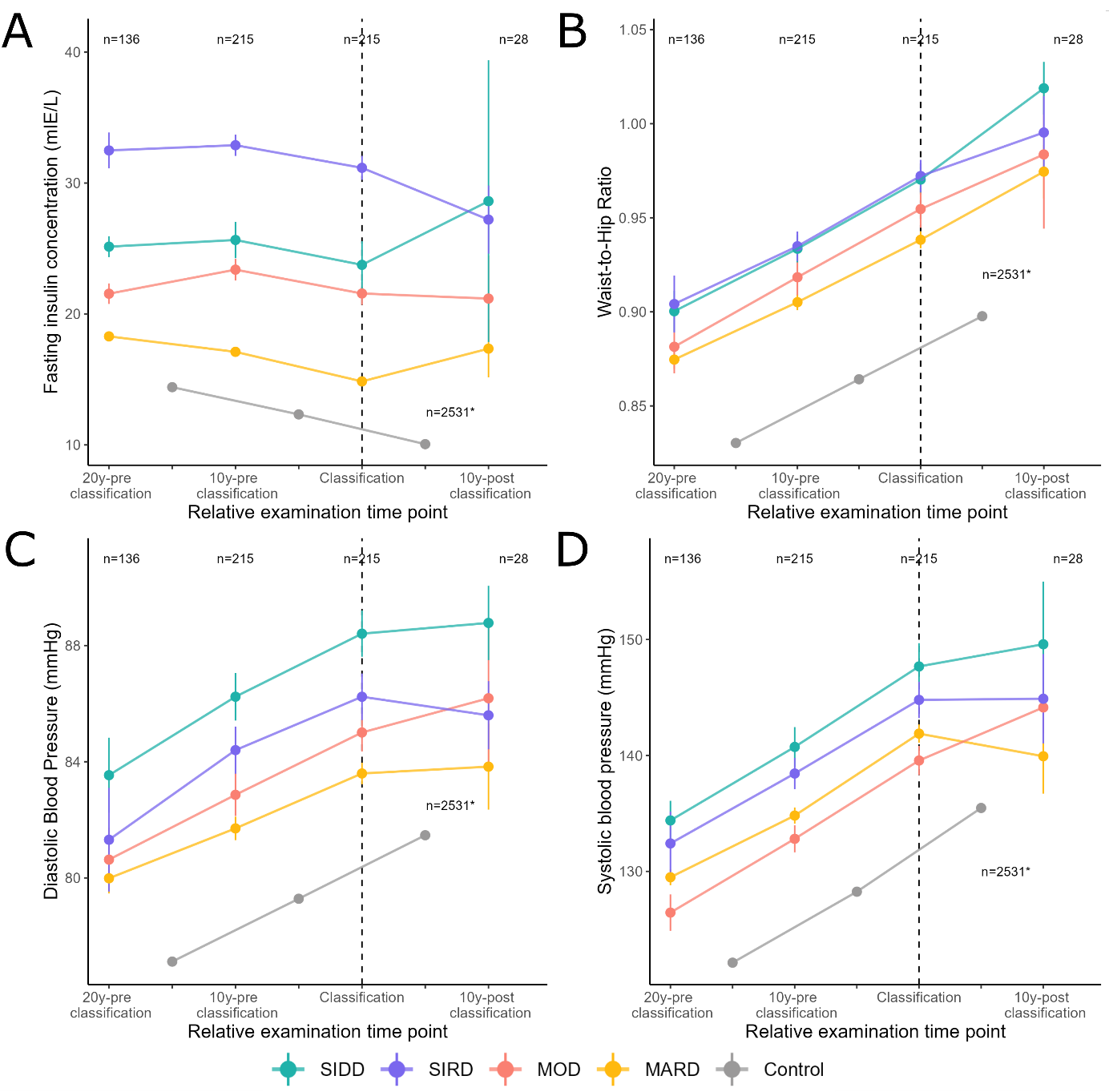
**

Multilevel longitudinal modelling was done for fasting insulin (A), waist-to-hip ratio (B), diastolic blood pressure (C) and systolic blood pressure (D) in individuals with incident diabetes (stratified by diabetes subtype) and in individuals without diabetes.

* n=2,548 for all examination time points of the control group.

Analysis was adjusted for age, sex and examination time point.

Graph shows mean and standard error for the fixed effects.

For the definitions of the time point of classification and the pre- and post-classification time periods, please see “Definition of the time point of classification and of the time periods before and after classification” in the methods section for details.

10y-pre classification, 10 years before classification; 20y-pre classification, 20 years before classification; 10y-post classification, 10 years after classification; HOMA2-B, homoeostatic model assessment 2 estimates of β-cell function; HOMA2-IR, homoeostatic model assessment 2 estimates of insulin resistance; MARD, moderate age-related diabetes; MOD, moderate obesity-related diabetes; SIDD, severe insulin-deficient diabetes; SIRD, severe insulin-resistant diabetes; T2D, type 2 diabetes.

**Supplementary Table 2** P-values for pairwise comparisons, model 1 and 2

| **Fasting glucose** | | | | | |
| --- | --- | --- | --- | --- | --- |
|  | Control | SIDD | SIRD | MOD | MARD |
| Control | - | <.0001 | <.0001 | <.0001 | <.0001 |
| SIDD | <.0001 | - | <.0001 | <.0001 | <.0001 |
| SIRD | <.0001 | <.0001 | - | 0.0001 | 1.0000 |
| MOD | <.0001 | <.0001 | 0.0001 | - | <.0001 |
| MARD | <.0001 | <.0001 | 0.4963 | 0.0029 | - |
| **BMI** | | | | | |
|  | Control | SIDD | SIRD | MOD | MARD |
| Control | - | <.0001 | <.0001 | <.0001 | <.0001 |
| SIDD | <.0001 | - | 0.0434 | 0.0025 | 0.0045 |
| SIRD | <.0001 | 0.0311 | - | 1.0000 | <.0001 |
| MOD | <.0001 | 0.0020 | 1.0000 | - | <.0001 |
| MARD | <.0001 | 0.0059 | <.0001 | <.0001 | - |
| **HOMA2-B** | | | | | |
|  | Control | SIDD | SIRD | MOD | MARD |
| Control | - | 0.0508 | <.0001 | 0.0812 | <.0001 |
| SIDD | 0.0002 | - | <.0001 | 1.0000 | 1.0000 |
| SIRD | 0.5024 | <.0001 | - | <.0001 | <.0001 |
| MOD | <.0001 | 1.0000 | <.0001 | - | 1.0000 |
| MARD | <.0001 | 1.0000 | <.0001 | 1.0000 | - |
| **HOMA2-IR** | | | | | |
|  | Control | SIDD | SIRD | MOD | MARD |
| Control | - | <.0001 | <.0001 | <.0001 | <.0001 |
| SIDD | <.0001 | - | <.0001 | 1.0000 | <.0001 |
| SIRD | <.0001 | 0.0160 | - | <.0001 | <.0001 |
| MOD | <.0001 | 0.0009 | <.0001 | - | <.0001 |
| MARD | <.0001 | <.0001 | <.0001 | 1.0000 | - |

Data are P-values from pairwise comparisons of the estimated marginal means, Bonferroni adjusted for number of pairwise comparisons made between subtypes. Results of model 1 are shaded grey. Unshaded rows correspond to results from model 2.

HOMA2-B, homoeostatic model assessment 2 estimates of β-cell function; HOMA2-IR, homoeostatic model assessment 2 estimates of insulin resistance; MARD, moderate age-related diabetes; MOD, moderate obesity-related diabetes; SIDD, severe insulin-deficient diabetes; SIRD, severe insulin-resistant diabetes.

**Supplementary Table 3** P-values for pairwise comparisons, model 1 and 2

| **Fasting insulin** | | | | | |
| --- | --- | --- | --- | --- | --- |
|  | Control | SIDD | SIRD | MOD | MARD |
| Control | - | <.0001 | <.0001 | <.0001 | <.0001 |
| SIDD | <.0001 | - | <.0001 | 1.0000 | <.0001 |
| SIRD | <.0001 | 0.0009 | - | <.0001 | <.0001 |
| MOD | <.0001 | 0.0024 | <.0001 | - | <.0001 |
| MARD | <.0001 | <.0001 | <.0001 | 1.0000 | - |
| **Waist-to-Hip Ratio** | | | | | |
|  | Control | SIDD | SIRD | MOD | MARD |
| Control | - | <.0001 | <.0001 | <.0001 | <.0001 |
| SIDD | 0.1678 | - | 1.0000 | 1.0000 | 1.0000 |
| SIRD | 0.1394 | 1.0000 | - | 1.0000 | 0.0009 |
| MOD | 1.0000 | 1.0000 | 1.0000 | - | 0.0149 |
| MARD | <.0001 | 1.0000 | 1.0000 | 0.1147 | - |
| **Diastolic Blood Pressure** | | | | | |
|  | Control | SIDD | SIRD | MOD | MARD |
| Control | - | 0.0001 | 0.0001 | 0.0063 | 0.0014 |
| SIDD | 0.0452 | - | 1.0000 | 1.0000 | 0.0943 |
| SIRD | 1.0000 | 1.0000 | - | 1.0000 | 0.5380 |
| MOD | 1.0000 | 0.2571 | 1.0000 | - | 1.0000 |
| MARD | 0.0846 | 1.0000 | 1.0000 | 1.0000 | - |
| **Systolic Blood Pressure** | | | | | |
|  | Control | SIDD | SIRD | MOD | MARD |
| Control | - | <.0001 | <.0001 | 0.0004 | <.0001 |
| SIDD | 0.0008 | - | 1.0000 | 0.7160 | 0.0560 |
| SIRD | 0.2201 | 1.0000 | - | 1.0000 | 0.2757 |
| MOD | 1.0000 | 0.0307 | 1.0000 | - | 1.0000 |
| MARD | <.0001 | 0.8972 | 1.0000 | 0.3300 | - |

Data are P-values from pairwise comparisons of the estimated marginal means, Bonferroni adjusted for number of pairwise comparisons made between subtypes. The results of model 1 are shaded grey. Unshaded rows correspond to results from model 4.

MARD, moderate age-related diabetes; MOD, moderate obesity-related diabetes; SIDD, severe insulin-deficient diabetes; SIRD, severe insulin-resistant diabetes.

**Supplementary Figure 5-** Trajectories of variables defining the diabetes subtypes (classification variables), Sensitivity analysis, Control sample without family history of diabetes

**
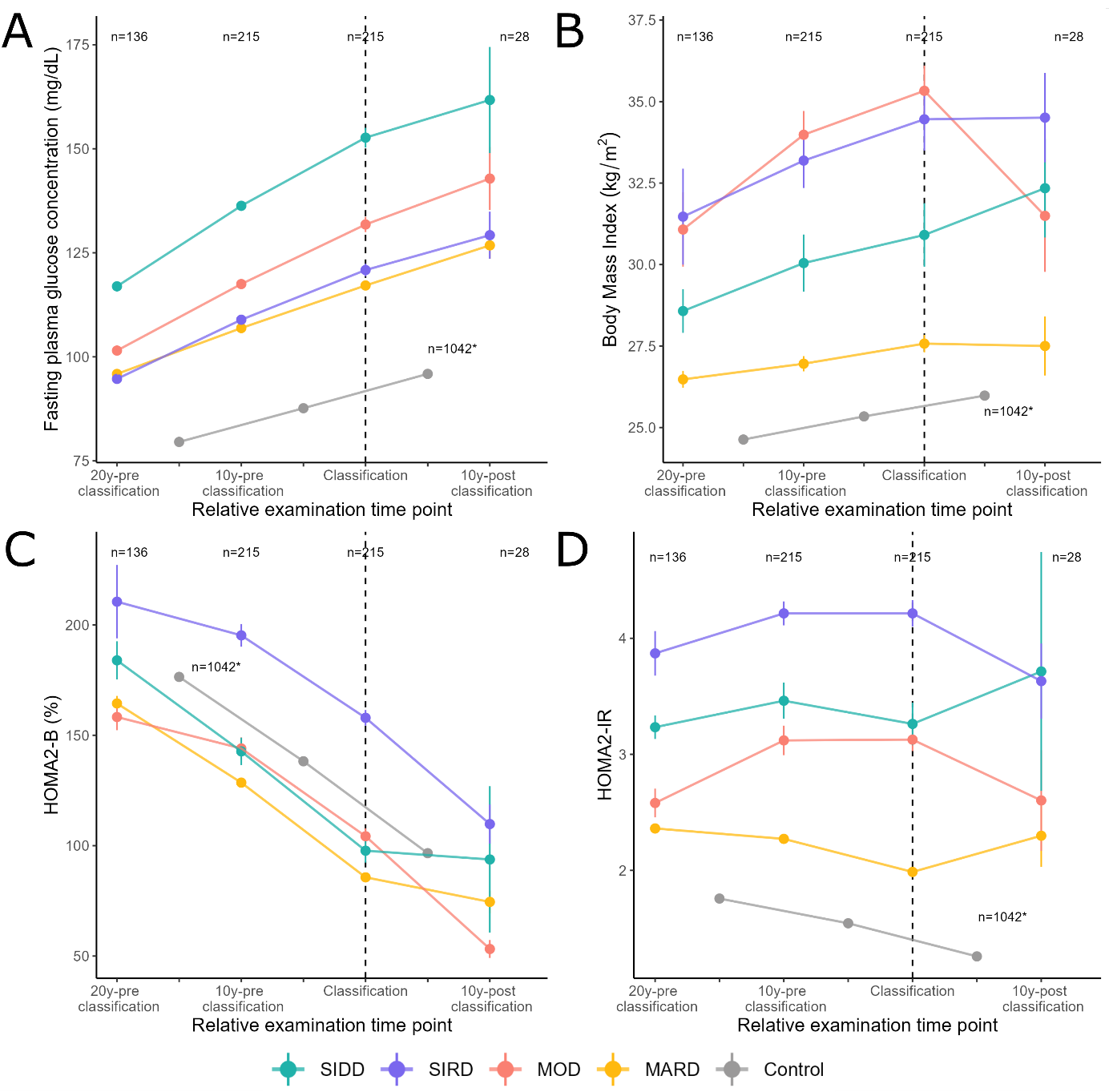
**

Multilevel longitudinal modelling was done for fasting glucose (A), body mass index (B), HOMA2-B (C), HOMA2-IR (D) in individuals with incident diabetes (stratified by diabetes subtype) and in individuals without diabetes

* n=1,042 for all examination time points of the control sample.

Graphs show mean and standard error for the fixed effects.

The sensitivity analysis model was adjusted for age, sex, study time point, education, body mass index (except for models with body mass index as the dependent variable), smoking behaviour, family history of diabetes, physical activity, and blood pressure medication (only for models with systolic and diastolic blood pressure as the dependent variables).

For the definitions of the time point of classification and the pre- and post-classification time periods, please see “Definition of the time point of classification and of the time periods before and after classification” in the methods section for details.

10y-pre classification, 10 years before classification; 20y-pre classification, 20 years before classification; 10y-post classification, 10 years after classification; HOMA2-B, homoeostatic model assessment 2 estimates of β-cell function; HOMA2-IR, homoeostatic model assessment 2 estimates of insulin resistance; MARD, moderate age-related diabetes; MOD, moderate obesity-related diabetes; SIDD, severe insulin-deficient diabetes; SIRD, severe insulin-resistant diabetes; T2D, type 2 diabetes.

**Supplementary Figure 6-** Trajectories of additional diabetes-related health parameters, Sensitivity analysis, Control sample without family history of diabetes


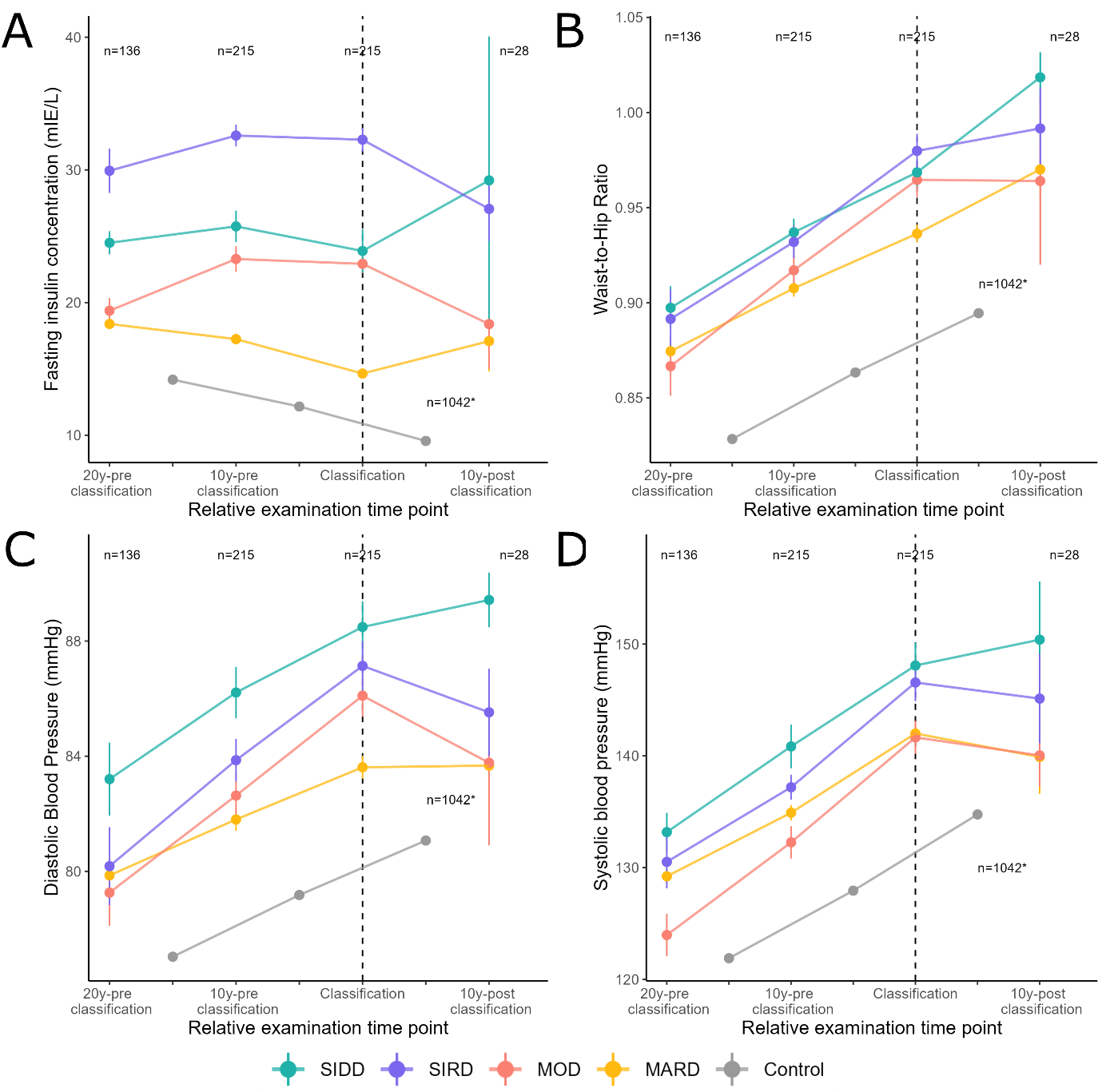


Multilevel longitudinal modelling was done for fasting insulin (A), waist-to-hip ratio (B), diastolic blood pressure (C), systolic blood pressure (D) in individuals with incident diabetes (stratified by diabetes subtype) and in individuals without diabetes.

* n=1,042 for all examination time points of the control group.

Graphs show mean and standard error for the fixed effects.

The sensitivity analysis model was adjusted for age, sex, study time point, education, body mass index (except for models with body mass index as the dependent variable), smoking behaviour, family history of diabetes, physical activity, and blood pressure medication (only for models with systolic and diastolic blood pressure as the dependent variables).

For the definitions of the time point of classification and the pre- and post-classification time periods, please see “Definition of the time point of classification and of the time periods before and after classification” in the methods section for details.

10y-pre classification, 10 years before classification; 20y-pre classification, 20 years before classification; 10y-post classification, 10 years after classification; HOMA2-B, homoeostatic model assessment 2 estimates of β-cell function; HOMA2-IR, homoeostatic model assessment 2 estimates of insulin resistance; MARD, moderate age-related diabetes; MOD, moderate obesity-related diabetes; SIDD, severe insulin-deficient diabetes; SIRD, severe insulin-resistant diabetes; T2D, type 2 diabetes.

**Supplementary Table 4-** β-coefficients for the trajectories of variables defining the diabetes subtypes (classification variables), fully adjusted model 2

|  | **β** | | | |
| --- | --- | --- | --- | --- |
| **Subtype** | **Fasting glucose** | **BMI** | **HOMA2-B** | **HOMA2-IR** |
| SIDD | 42.51 | 4.22 | -34.73 | 1.22 |
| SIRD | 16.03 | 6.94 | 12.96 | 1.69 |
| MOD | 24.62 | 7.70 | -39.57 | 0.63 |
| MARD | 19.05 | 1.56 | -26.95 | 0.50 |

**Supplementary Table 5-** β-coefficients for the trajectories of additional diabetes-related health parameters, fully adjusted model 2

|  | **β** | | | |
| --- | --- | --- | --- | --- |
| **Subtype** | **Fasting insulin** | **Waist-to-Hip Ratio** | **Diastolic Blood Pressure** | **Systolic Blood Pressure** |
| SIDD | 8.27 | 0.02 | 4.41 | 9.62 |
| SIRD | 12.89 | 0.02 | 1.22 | 4.61 |
| MOD | 3.85 | 0.004 | -0.08 | 0.24 |
| MARD | 3.20 | 0.02 | 1.81 | 5.19 |

**Supplementary Table 6-** β-coefficients for the trajectories of variables defining the diabetes subtypes (classification variables), Model 1

|  | **β** | | | |
| --- | --- | --- | --- | --- |
| **Subtype** | **Fasting glucose** | **BMI** | **HOMA2-B** | **HOMA2-IR** |
| SIDD | 43.76 | 4.52 | -23.13 | 1.56 |
| SIRD | 20.98 | 7.17 | 32.83 | 2.32 |
| MOD | 29.48 | 7.98 | -18.49 | 1.31 |
| MARD | 20.17 | 1.77 | -23.53 | 0.64 |

**Supplementary Table 7-** β-coefficients for the trajectories of additional diabetes-related health parameters, Model 1

|  | **β** | | | |
| --- | --- | --- | --- | --- |
| **Subtype** | **Fasting insulin** | **Waist-to-Hip Ratio** | **Diastolic Blood Pressure** | **Systolic Blood Pressure** |
| SIDD | 10.95 | 0.05 | 7.15 | 15.05 |
| SIRD | 17.89 | 0.07 | 5.54 | 12.68 |
| MOD | 9.19 | 0.07 | 4.61 | 8.99 |
| MARD | 4.30 | 0.04 | 2.68 | 7.37 |
